# Efficacy and Safety of Elexacaftor-Tezacaftor-Ivacaftor in the Treatment of Cystic Fibrosis: A Systematic Review

**DOI:** 10.3390/children10030554

**Published:** 2023-03-15

**Authors:** Nikoletta Kapouni, Maria Moustaki, Konstantinos Douros, Ioanna Loukou

**Affiliations:** 1 Pediatric Allergy and Respiratory Unit, 3rd Department of Pediatrics, “Attikon” University Hospital, National and Kapodistrian University of Athens, School of Medicine, 11527 Athens, Greece; nikolkap_naxos@hotmail.com (N.K.); costasdouros@gmail.com (K.D.); 2 Cystic Fibrosis Department, “Agia Sofia” Children’s Hospital, 11527 Athens, Greece; mar.moustaki@gmail.com

**Keywords:** cystic fibrosis, cystic fibrosis transmembrane conductance regulator (CFTR) protein, CFTR modulators, elexacaftor, ivacaftor, tezacaftor, kaftrio, trikafta

## Abstract

Elexacaftor/Tezacaftor/Ivacaftor (ELX/TEZ/IVA) is a new CFTR (Cystic Fibrosis Transmembrane Conductance Regulator) modulator treatment, used over the last few years, which has shown an improvement in different clinical outcomes in patients with cystic fibrosis (CF). The objective of this study was a systematic research of the literature on the efficacy and safety of this CFTR modulator on patients with CF. A search of Pubmed was conducted for randomized clinical trials and observational studies published from 2012 to September 2022. The included full manuscripts comprised nine clinical trials and 16 observational studies, whose participants were aged ≥12 years or were children 6–11 years old with at least one Phe508del mutation and/or advanced lung disease (ALD). These studies reported that ELX/TEZ/IVA has a significant positive effect on the lung function of patients with CF, by ameliorating parameters such as FEV_1_, LCI, pulmonary exacerbations or sweat chloride concentration, increasing BMI and improving quality of their life. Its role in cystic fibrosis-related diabetes (CFRD) is not yet clear. It was found that this new CFTR modulator has an overall favorable safety profile, with mild to moderate adverse events. Further studies are needed for a deeper understanding of the impact of CFTR modulators on other CF manifestations, or the possibility of treating with ELX/TEZ/IVA CF patients with rare CFTR mutations.

## 1. Introduction

Cystic fibrosis (CF) is an autosomal recessive genetic disease, which affects approximately 70,000 people worldwide [1]. It is caused by mutations of the Cystic Fibrosis Transmembrane Conductance Regulator (CFTR) gene, which encodes for the CFTR protein, located on the apical surface of epithelial cells of multiple tissues (respiratory system, gastrointestinal tract, male reproductive organs, pancreas, sweat glands, etc.) and is responsible for chloride and bicarbonate transport across the epithelial surfaces. Defective CFTR protein leads to a decrease in chloride secretion and an increase in sodium absorption, followed by an osmotic uptake of water, provoking thick fluid secretions. In addition, diminished bicarbonate secretion is responsible for inadequate alkalization of pH and, though impairment of antimicrobial factors, of the surface liquid. As a result, chronic pulmonary inflammation and infection develop and bronchiectasis and progressive lung function decline, in association with impaired gastrointestinal function (bowel obstruction, hepatobiliary disease and pancreatic insufficiency), malnutrition and infertility [2,3,4].

There are more than 2000 CFTR sequence variants and approximately 350 of them are known to cause CF. Phe508del mutation is the most common mutation and 88% of patients have at least one copy [2,3]. CFTR mutations are classified into six categories according to the molecular mechanism of decreased functional expression: in class I mutations, the production of CFTR protein is diminished; class II mutations lead to misfolding of CFTR protein, which is unable to reach the cell surface (including the Phe508del mutation); in class III mutations, the CFTR protein is not functional (“gating mutations”–for example G551D mutation); in class IV mutations. ions transport is diminished; class V mutations produce inadequate quantities of the CFTR protein and class VI mutations produce a less stable CFTR protein. Class I-III mutations cause a more severe form of the disease, whereas class IV-VI mutations lead to a milder CF phenotype [2,3].

Until recently the target of CF treatment was the management of symptoms (including airway clearance, antibiotics, and nutritional support). During the last decade, new drugs were developed that target the underlying defective CFTR protein. These drugs, named CFTR modulators, are split into two categories: correctors, which are small molecules improving the structure and trafficking of the defective CFTR protein, and potentiators, which prolong the period the CFTR protein channel remains open, and so increase chloride transport [5,6]. Ivacaftor (IVA), the first CFTR modulator approved by the FDA in 2012, is a potentiator used originally for the treatment of patients with CF and at least one G551D; since then, it has been approved for more mutations. However, it is not effective for patients carrying the most common mutation, Phe508del, since very little protein is expressed and ivacaftor does not impact expression. The CFTR corrector Lumacaftor (LUM) is used to improve the transport of the CFTR protein on the cell surface. The combination Lumacaftor-Ivacaftor is used for Phe508del homozygous patients. The next corrector Tezacaftor, paired with Ivacaftor, has sufficient effect on patients who are Phe508del homozygous or those with one Phe508del copy and one residual mutation (a CFTR mutation that causes less important damage to CFTR protein function).

The triple combination Elexacaftor (a new corrector)-Tezacaftor-Ivacaftor (Trikafta or Kaftrio) leads to clinical improvements in those with one or two copies of the Phe508del variant; it works even for those who are Phe508del heterozygous, with a minimal function mutation, defined as the complete absence of CFTR production, or lack of in-vitro responsiveness to Ivacaftor/Tezacaftor [6]. This triple combination was first approved in the USA in October 2019 for patients ≥ 12 years old [7], but has become available for children ≥ 6 years old since June 2021. In the European Union, it was approved in 2021 for patients aged ≥ 12 years and has become available for children ≥ 6 years old since January 2022. It is of interest also to notice that, according to the recent prescribing information for the medication, it is indicated for responsive CFTR mutations based on in vitro data.

In the clinical trials of triple combination therapy, CF patients have shown improvements and amelioration of their quality of life. The treatment was also well-tolerated with mostly moderate adverse events. The purpose of this systematic review is to summarize the efficacy and effectiveness of the Elexacaftor/Tezacaftor/Ivacaftor combination in the treatment of people with CF, as well as to report on the safety of this medication.

## 2. Materials and Methods

### 2.1. Literature Search, Eligibility Criteria and Study Selection

This systematic review consists of two different kinds of studies; the first part includes clinical trials and the second observational studies.

The selection of the clinical trials was performed following the PRISMA (Preferred Reporting Items for Systematic reviews and Meta-Analyses) guidelines [8]. Eligible studies were reported in PubMed, up to the data collection time (September 2022). Only studies published in the English language were selected.

Study characteristics were framed by using the PICO criteria to include the population (P), intervention (I), comparison (C) and outcome (O). The studies reviewed must have examined people with CF, Phe508del homozygous or heterozygous, as the target group and the intervention must have been the use of the CFTR modulator Elexacaftor/Tezacaftor/Ivacaftor. The studies mentioned must have had a comparison group, as the review concerns randomized controlled trials, and must report at least one relevant endpoint, more precisely, changes in percentage predicted forced expiratory volume in the first second (ppFEV_1_), sweat chloride concentration (SCC), CFQ-R respiratory domain score (CFQ-R RD), body mass index (BMI), exacerbation rates, healthcare visits, and type and frequency of adverse events due to the treatment.

As far as observational studies are concerned, they must have included CF patients with Phe508del/Phe508del or Phe508del/Minimal Function genotypes, treated with triple combination therapy (Elexacaftor/Tezacaftor/Ivacaftor), controlled for a specific period of time and reporting at least one outcome related to our study (pulmonary function, BMI, CFQR, sweat chloride concentration, exacerbation rates, healthcare visits, glycemic status, adverse events of treatment).

### 2.2. Study Selection

**Figure 1:**
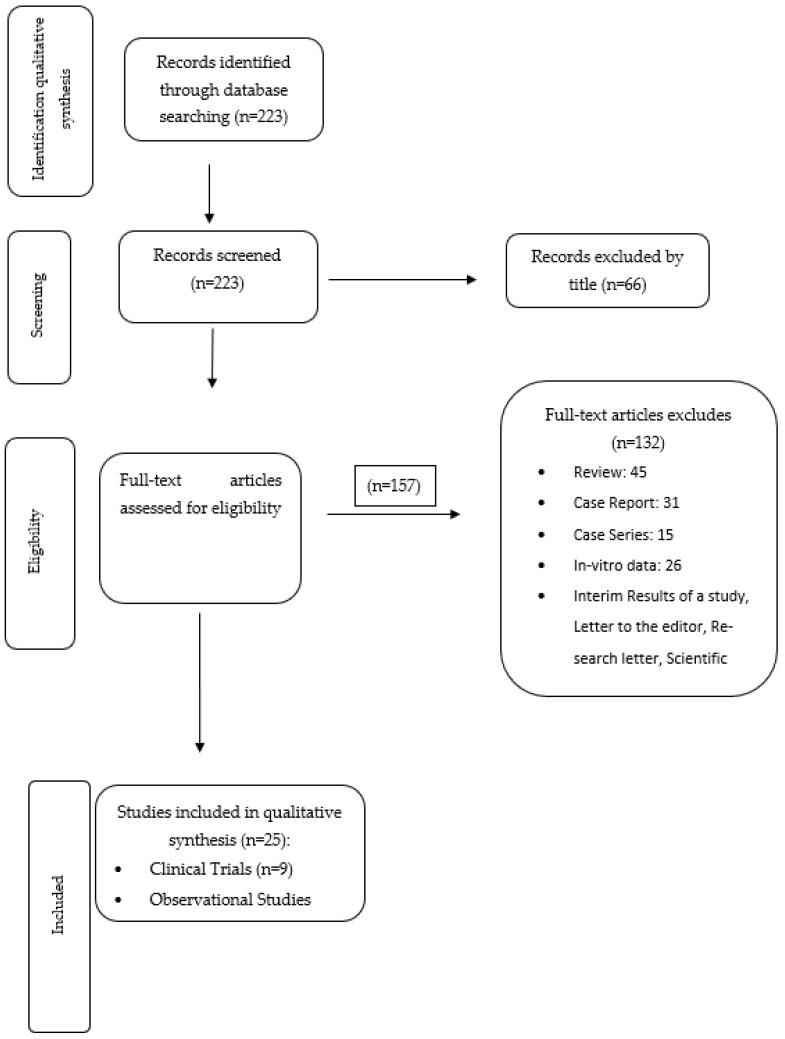
Flow diagram of study selection.

This systematic search initially yielded 223 papers, all of which were published in the last decade, indicative of the recent development of these drugs. The keywords used to conduct the search were elexacaftor, tezacaftor, ivacaftor. Data were extracted by two researchers, who reviewed the search results independently. Any disagreements were resolved by consensus and discussion. The review was based on titles and abstracts and when the abstract was not helpful the full manuscript was reviewed. 223 articles were screened, 66 of which were excluded by title, and 157 were screened for eligibility. Finally, 25 articles met the eligibility criteria for inclusion (nine clinical trials and 16 observational studies). Figure 1 depicts the various steps of the data collection and selection process.

## 3. Results

### 3.1. Data from Case-Control Studies

#### 3.1.1. Efficacy of Elexacaftor-Tezacaftor-Ivacaftor

**Pulmonary Efficacy and effect on sweat chloride concentration**

The most commonly used indicators of lung function in CF patients are the ppFEV_1_ and the occurrence of pulmonary exacerbations (PEx). Another important tool is the Cystic Fibrosis Questionnaire-Revised Respiratory Domain (CFQ-R RD), which is a measure of subjective amelioration of pulmonary symptoms and quality of life. An alternative research measure of lung health is the Lung Clearance Index (LCI), usually reported as LCI_2.5_, which is a measure of ventilation inhomogeneity [7].

Keating et al., in a phase 2 clinical trial with adult patients with at least one Phe508del copy, treated with Elexacaftor/Tezacaftor/Ivacaftor for 4 weeks, revealed in patients with Phe508del/Minimal function (MF) genotype (n = 95) an increase of ppFEV_1_ up to 13.8 points (95% CI 10.9–16.6. *p* < 0.001), an absolute change in sweat chloride concentration (SCC) of −39.1 mmol/lt (95% CI −44.9 to −33.3), and amelioration of CFQ-R RD score by 25.7 points (95% CI 18.3–33.1). Patients with Phe508del/Phe508del genotype (n = 24) showed an 11% (95% CI 7.9–14.0, *p* < 0.001) improvement in ppFEV_1_, SCC was diminished by 39.6 mmol/lt (95% CI −45.3 to −33.8), and CFQ-R RD score increased by 20.7 points (95% CI 12.5–29), relative to control group [9]. In addition, Middleton et al., in a phase 3 clinical trial, included 403 patients ≥12 years old with Phe508del/MF genotype and ppFEV1 40 to 90% receiving the triple combination therapy, and observed an improvement in ppFEV_1_ of 13.8 points (95% CI 12.4–15.4, *p* < 0.001) and 14.3 points (*p* < 0.001) from baseline relative to placebo at week 4 and 24 respectively. Moreover, the results of the study showed a reduction of 63% of the annual rate of pulmonary exacerbations relative to placebo (Rate Ratio 0.37, 95% CI 0.25–0.55, *p* < 0.001). The SSC reduced by 41.8 mmol/lt (95% CI −44.4 to −39.3, *p* < 0.001) and the CFQ-R RD score increased by 20.2 points (95% CI 17.5–23.0, *p* < 0.001) through to week 24 compared to the placebo group [10].

The phase 3 trial, conducted by Heijerman et al., involving 113 patients aged ≥12 years, Phe508del homozygous, with ppFEV1 between 40–90% treated with ELX/TEZ/IVA after a 4-week period with TEZ/IVA, showed an increase in ppFEV_1_ of 10 points (95% CI 7.4–12.6, *p* < 0.001), a decrease in SCC with a mean treatment difference of −45.1 mmol/lt (95% CI −50.1 to −40.1, *p* < 0.001), and an improvement in CFQ-R RD score by 17.4 points at week 4 compared with patients receiving TEZ/IVA treatment [11]. Similarly, Sutharsan et al. designed a phase 3b trial using the same eligibility criteria for patients as Heijerman %et al. (patients ≥12 years old, Phe508del homozygous, with ppFEV_1_ 40–90% with a 4-week run-in period, in which patients received TEZ/IVA). In this research, at week 24 the ppFEV_1_ in ELX/TEZ/IVA group increased by 10.2 points (95% CI 8.2–12.1, *p* < 0.0001), the SCC decreased by 42.8 mmol/lt (95% CI −46.2 to −39.3, *p* < 0.0001), and the CFQ-R RD score augmented by 15.9 points (95% CI 11.7–20.1, *p* < 0.0001) relative to TEZ/IVA group [12].

Migliorisi et al., using a small sample of patients (n = 26) with at least one Phe508del copy (Phe508del/Phe508del or Phe508del/MF genotypes) found out that, in one year, the ppFEV_1_ of patients treated with the triple combination was augmented by 10–15 points, the number of pulmonary exacerbation was statistically significantly reduced (*p* < 0.05), 77% of the cases reported a diminished SCC and 100% of the cases showed an increase in the CFQ-R RD score [13]. Studying a different group in a phase 3 trial, namely 258 patients aged ≥12 years, with Phe508del/Gating mutation or Phe508del/Residual mutation genotypes, after a 4-week run-in period where they received IVA or IVA/TEZ, respectively, Barry et al. observed an increase by 3.5% (95% CI 2.2–4.7, *p* < 0.001) in ppFEV_1_, an absolute change in SCC by 23.1 mmol/lt (95% CI −26.1 to −20.2, *p* < 0.001), and an amelioration of CFQ-R RD score by 8.7 points (95% CI 5.3–12.1) through to week 8 with ELX/TEZ/IVA treatment compared to active control. Studying the role of genotype, the researchers assessed that, for patients with Phe508del/Gating mutation genotype, through to week 8 the mean increase in ppFEV_1_ was 5.8 points (95% CI 3.5–8.0), the SCC diminished by 20 mmol/lt (95% CI −25.4 to −14.6) and the CFQ-R RD score increased by 8.9 points (95% CI 3.8–14.0) relative to active control. For patients with Phe508del/Residual mutation genotype through to week 8, the mean change in ppFEV_1_ was 2% (95% CI 0.5–3.4), the mean change in SCC was −24.8 mmol/lt (95% CI −28.4 to −21.2), and the CFQ-R RD score was ameliorated by 8.5 points (95% CI 4.0–13.0) compared to the active control group [14].

In a phase 3b trial, conducted by Mall et al. with 121 young patients aged 6–11 years with Phe508del/MF genotypes, for subjects treated with ELX/TEZ/IVA the mean baseline LCI_25_ was 10.26 units and the mean ppFEV_1_ was 91.4%. After 24 weeks of treatment, there was a significant decrease in LCI_2.5_ of 2.26 units (95% CI −2.71 to −1.81, *p* < 0.0001) and an increase in ppFEV_1_ of 11 percentage points (95% CI 6.9–15.1, *p* < 0.001) for ELX/TEZ/IVA group versus placebo. The SCC in ELX/TEZ/IVA group diminished by 52.1 mmol/lt (95% CI −55 to −49.2) compared with a change of −0.9 mmol/lt (95% CI −3.8 to 2.0) in placebo group through week 24 [15]. Finally, Mainz et al. in a prospective study with 107 patients with at least one Phe508del mutation, as secondary endpoints observed that ppFEV_1_ in those treated with ELX/TEZ/IVA for 24 weeks increased by 13 percentage points compared to baseline (*p* < 0.0001) [16]. 

**Growth and Nutrition**

CFTR modulators have been effective in weight gain and BMI increase. Change in BMI was one of the secondary endpoints of the study conducted by Middleton et al., which noticed that patients ≥12 years receiving ELX/TEZ/IVA for 4 weeks experienced a BMI increase, with a mean treatment difference of 1.04 relative to the placebo group (95% CI 0.85–1.23, *p* < 0.001) [10]. The phase 3 study by Heijerman et al. revealed that Phe508del homozygous patients ≥12 years treated with ELX/TEZ/IVA had an improvement of BMI at week 4 of 0.60 kg/m^2^ (95% CI 0.41–0.79, *p* < 0.0001) and a mean body increase of 1.6 kg (95% CI 1–2.1, *p* < 0.0001) compared with TEZ/IVA group [11]. In addition, Migliorisi et al. found out that patients ≥ 12 years old, with at least one Phe508del mutation treated with ELX/TEZ/IVA for one year, significantly increased their BMI, compared to the control group [13]. Furthermore, Mainz et al., in a prospective study with 107 patients, noticed that for children treated with ELX/TEZ/IVA the means for BMI-for-age z-scores increased at week 22 from −0.71 ± 0.19 to −0.29 ± 0.24 (*p* = 0.002) and the mean weight from 47 ± 2.1 kg to 51.4 ± 2.3 kg (*p* < 0.0001). In adults the mean BMI and weight increased by 8% compared to baseline, from 22.2 ± 0.3 kg/m^2^ to 24 ± 0.4 kg/m^2^ (*p* < 0.0001) and from 63.1 ± 1.3 kg to 68.2 ± 1.4 kg (*p* < 0.0001), respectively [16].

**Other clinical parameters**

CF patients cope with multiple infections of the respiratory tract, which require frequent antibiotic use or even hospital admission. Miller et al. performed a case-crossover analysis which included 389 CF patients who began treatment with ELX/TEZ/IVA for a 15-week period and compared them with patients without treatment for the same period. The triple combination therapy revealed a decrease in the number of healthcare visits, −2.5 (95% CI −3.31 to −1.7), a change in patients’ admissions, −0.16 (95% CI −0.22 to −0.1), a diminished number of visits related with infections, −0.62 (95% CI −0.93 to −0.31) and a change in distinct antibiotics prescribed, −0.78 (95% CI −1.03 to 0.54). Because of the small sample of rare infections, the type of infections decreased was not mentioned, but *P. aeruginosa* and non-tuberculous mycobacterial infections were diminished. In addition, the secondary endpoints of the study showed a decrease in healthcare visits in different places by 3.51 (95% CI −4.55 to −2.47), a change in the number of days with an outpatient antibiotic supply, −0.78 (95% CI −0.81 to −0.43) and a change in days of antibiotic use, −16.08 (95% CI −22.5 to −9.66) [17].

Migliorisi et al. recorded the microbiological data from the sputum samples of CF patients in the ELX/TEZ/IVA group and the control group. After one year of the triple combination treatment, the colonization rates of respiratory samples progressively decreased and almost 45.3% of the sputum samples of patients in the ELX/TEZ/IVA group became negative for pathogenic bacteria [13].

CF patients suffer from gastrointestinal symptoms such as abdominal pain, bloating or nausea, so Mainz et al. conducted a multi-center prospective study with 152 participants aiming to assess any differences in abdominal symptoms. They used the CFAbd-Score in patients treated with ELX/TEZ/IVA treatment and compared them to healthy controls. The CFAbd-Score consists of five domains: pain, gastro-esophageal reflux disease (GERD), quality of life impairment (QoL), disorders of bowel movement (DBM) and disorders of appetite (DA). After 24 weeks in the triple combination treatment, the total CFAbd-Score decreased significantly by 19%, the pain by 22%, the GERD by 20%, the DBM by 12%, the QoL impairment by 28% and the DA by 39% (all *p* < 0.05). However, in comparison to healthy controls, the total CFAbd-Score and the following domains, pain, GERD, DA, QoL, of PwCF during the ELX/TEZ/IVA treatment showed no significant differences. On the other hand, the DBM domain was significantly elevated during the triple therapy compared to the control group (17.6 ± 1.3 and 11.9 ± 1.4, *p* = 0.004, respectively). Meteorism and abdominal pain were the two most common symptoms that decreased from 60% to 50% and 59% to 39%, respectively, after triple treatment initiation [16].

#### 3.1.2. Safety of Elexacaftor-Tezacaftor-Ivacaftor

The safety of the triple combination treatment was assessed by Keating et al. In their study, 92% of patients in the ELX/TEZ/IVA group, 100% of people in the placebo group and 71% of patients in TEZ/IVA group reported at least one adverse event (AE). Patients who received ELX/TEZ/IVA had mild events (53%), moderate events (43%) and serious events (4%). The most frequent side effects observed in patients who received the triple therapy were cough, increased sputum production, pulmonary exacerbations, hemoptysis and pyrexia. Elevated levels of aminotransferase greater than three times the upper limit occurred in 8%, and elevated bilirubin levels greater than two times the upper limit occurred in 3% of patients in this group. Concerning the serious side effects, cases of infective pulmonary exacerbation, distal intestinal obstruction syndrome and jugular venous thrombosis were noticed. No deaths occurred, but side effects such as rash, elevated bilirubin levels and chest pain, that occurred in three patients in ELX/TEZ/IVA group, led to the discontinuation of the trial. The administration of the triple combination was stopped in three patients because of adverse effects: elevated levels of SGOT, SGPT, CPK and myopathy in the first; elevated levels of bilirubin in the second; and constipation in the third [9].

Middleton et al. described the AE that occurred during their study, in which 93.1% of patients in the ELX/TEZ/IV group showed at least one, compared to 96% in the placebo group. The majority of patients in the ELX/TEZ/IVA group had mild (33.2%) and moderate (50.5%) AE and only 13.9% had a serious AE (ex. infective pulmonary exacerbation) compared to the control group (20.9% with a serious AE). In previous studies for other CFTR modulator therapy, rash and elevated levels of aminotransferase were observed, so these specific side effects were also assessed in this trial. A total of 10.9% of patients in ELX/TEZ/IVA group had elevated aminotransferase levels, mostly greater than three times the upper normal range (7.9%), compared to 4% in the placebo group. As for rash, it occurred in 10.9% of patients in the ELX/TEZ/IVA group and 6.5% in the control group. Moreover, elevated CPK levels were noticed, mostly associated with exercise, and changes in blood pressure (systolic and diastolic blood pressure increased by 3.1 mmHg and 1.9 mmHg, respectively), at week 24. No deaths were observed, but two patients in ELX/TEZ/IVA group discontinued the trial because of a rash in one patient and portal hypertension in another with cirrhosis [10].

Heijerman et al. noted that ELX/TEZ/IVA was generally well tolerated in a 4-week trial. Side effects were seen in 58% of participants in the ELX/TEZ/IVA group and 33% in the TEZ/IVA group. The side effects were mostly mild and moderate; serious AE occurred in 4% of the patients in ELX/TEZ/IVA group (rash and pulmonary exacerbation) and 2% in TEZ/IVA group (pulmonary exacerbation). The most common side effects reported were cough (15% in ELX/TEZ/IVA group and 8% in TEZ/IVA group) and pulmonary exacerbation (2% and 12%, respectively). Elevated levels of SGOT and SGPT greater than three, five or eight times the upper normal range occurred in 7%, 4% and 0% of the participants in the triple combination group, respectively, compared with no aminotransferase elevations in the TEZ/IVA group. A mild rash was observed in 4% of participants in the ELX/TEZ/IVA group and 4% in the control group. No deaths and no discontinuation of the trial in either treatment group were reported [11].

In the trial conducted by Barry et al., 66.7% of the patients in the ELX/TEZ/IVA group and 65.9% of those in the active control group (receiving Ivacaftor or Ivacaftor-Tezacaftor treatment) had at least one AE, mostly mild or moderate in severity. Serious AE were noticed in 3.8% of patients in the ELX/TEZ/IVA group and 8.7% in the control group. Elevated levels of aminotransferase occurred in 6.1% of patients in the ELX/TEZ/IVA group and 0.8% in the control group, and CPK levels increased in 1.5% and no patients, respectively. Rash, mild or moderate in severity, was seen in 3% of patients in the triple combination group and 4% in the control group. As for the blood pressure, the mean systolic and the mean diastolic pressure increased by 3 mmHg and 2.5 mmHg in the ELX/TEZ/IVA group and 0.5 mmHg and 0.3 mmHg in the control group, respectively, without any cases of hypertension in any group. The investigators did not notice any deaths, but the treatment was discontinued in one patient in the ELX/TEZ/IVA group because of elevated SGOT and SGPT levels and in two patients in the active control group due to anxiety, depression and pulmonary exacerbation [14].

In the phase 3b trial, conducted by Sutharsan et al., 89% of patients in the ELX/TEZ/IVA group and 92% in the TEZ/IVA group had an AE at week 24 of treatment, with the majority being mild or moderate in severity. The most common AE in the ELX/TEZ/IVA group was headache (29%) and nasopharyngitis (20%), and 6% of patients in the triple combination therapy and 16% in TEZ/IVA group had serious side effects. No deaths occurred, but one participant in the ELX/TEZ/IVA group discontinued the treatment owing to anxiety and depression, and two patients in the active control group due to psychotic disorder and obsessive-compulsive disorder. Elevated SGOT and SGPT levels greater than three times the upper normal level were reported in 6% of patients in the ELX/TEZ/IVA group and 6% in the TEZ/IVA group. Rash was observed in 13% of participants in the triple therapy group and 2% in the active control group. Increased levels of CPK were seen in 5% of patients in the ELX/TEZ/IVA group and 2% in the TEZ/IVA group. None of these side effects were serious and no treatment interruption was needed [12].

Mall et al. studied the safety of ELX/TEZ/IVA in children with CF aged 6–11 years. 80% of children in the ELX/TEZ/IVA group and 93.4% in the placebo group had AE, mostly mild and moderate in severity. The most frequent AE in the ELX/TEZ/IVA group were headache (30%) and cough (23.3%), whereas in the control group cough (42.6%), abdominal pain (27.9%), infective pulmonary exacerbation (26.2%), headache (19.7%), and oropharyngeal pain (19.7%); 6.7% of children in the triple combination group and 14.8% in the placebo group had serious side effects. Among children in the ELX/TEZ/IVA group, elevated levels of SGOT and SGPT more than three times the upper limit of normal, were noticed in 13.6% of patients, more than five times in 5.1%, and more than eight times in 1.7%. Among children in the placebo group, 4.9%, 1.6% and 0% had aminotransferase levels more than three, five or eight times the upper normal range, respectively. Concerning the rashes, 13.3% of children in the ELX/TEZ/IVA group and 4.9% in the placebo group had eruptions, but only one child in the triple combination therapy group discontinued the trial due to a serious rash. AE of hypertension and CPK levels increase occurred in no children in either group [15].

The above mentioned results are summarized in Table 1. 

### 3.2. Data Based on Observational Studies

#### 3.2.1. Efficacy of Elexacaftor-Tezacaftor-Ivacaftor

**Pulmonary efficacy and effect on sweat chloride concentration**

DiMango et al., in a prospective cohort study with 43 patients with at least one copy of Phe508del mutation, noted that after 3 months of ELX/TEZ/IVA treatment the ppFEV_1_ increased from 65% to 76% (*p* < 0.001) and the CFQ-R RD score was significantly improved [18].

Nichols et al. conducted a prospective, observational study in 487 patients aged ≥12 years with at least one Phe508del mutation in order to assess the effectiveness of ELX/TEZ/IVA treatment after 6 months of therapy. At baseline, 50.9% of the participants did not receive any CFTR modulator therapy, 44.1% were on Lumacaftor/Ivacaftor (LUM/IVA) treatment and 6.7% were on Ivacaftor (IVA). At 6 months of triple treatment, the ppFEV_1_ increased in the whole cohort by 9.8 percentage points (95% CI 8.8–10.8). The greater increase was noticed in those without previous treatment (10.8, 95% CI 9.3–12.4) and with LUM/IVA therapy (9.2, 95% CI 7.8–10.7). For those under IVA treatment before the study, the average improvement was 6.1 (95% CI 3.3–8.9). Furthermore, after 6 months of ELX/TEZ/IVA therapy, the overall SCC change was −41.7 mmol/L (95% CI −43.8 to −39.6) with a greater improvement among those in LUM/IVA treatment (−43.3 mmol/L, 95% CI −46.4 to −40.4) and those with no previous treatment (−43.2, 95% CI −46.2 to −40.1). However, a significant decrease was also observed in the IVA group (−23.9 mmol/L, 95% CI −31 to −16.8). The CFQ-R RD score was ameliorated in the entire cohort, with a mean change of 20.4 points (95% CI 18.28–22.50) and a more important improvement among patients without previous treatment (22.51, 95% CI 19.47–25.54). It was noticed that the need for symptomatic treatment was limited, and 6.0% fewer patients used dornase alfa, 9.8% hypertonic saline, 9.1% azithromycin and 34% inhaled antibiotics, relative to baseline, after 6 months of treatment [19].

Graeber et al. included 107 patients (55 Phe508del/MF genotype and 52 Phe508del homozygous) ≥12 years old in a prospective observational, multicenter study in order to assess the effects of ELX/TEZ/IVA treatment 8–16 weeks after initiation. The results showed that Phe508del/MF patients after ELX/TEZ/IVA treatment had a ppFEV_1_ increase by 13 points (interquartile range (IQR): 7.1–21.5, *p* < 0.001) and a SCC decrease by 48.5 mmol/L (IQR: −65.3 to −34.1, *p* < 0,001) from baseline. ELX/TEZ/IVA treatment led to 8.4 points (IQR: 2.7–15.7, *p* < 0.001) and 10.5 points (IQR: 5–15.3, *p* < 0.001) increase in patients Phe508del homozygous, pretreated with TEZ/IVA and those without previous treatment, respectively. SCC of participants with Phe508del/Phe508del genotype was reduced by 50.5 mmol/L (IQR −60.3 to −36.3, *p* < 0.001), and by 61 mmol/L (IQR: −74 to −41, *p* < 0.001) among patients with previous two-drug therapy and those without treatment, respectively [20].

In a prospective, observational, multicenter study conducted by Graeber et al., 91 CF patients, aged ≥12 years (45 with Phe508del/MF and 46 with Phe508del/Phe508del genotype) participated. They received an ELX/TEZ/IVA treatment for 8–16 weeks. Patients with Phe508del/MF had LCI at baseline at 10.3 (IQR: 8–13.2), which improved to 7.4 (IQR: 6.5–10.3, *p* < 0.001) after the triple treatment, reflecting a relative increase of 23.4%; the ppFEV_1_ increased by 14.5 points (IQR: 8–24, *p* < 0.001); SCC decreased by a median of −49 mmol/lt (IQR: −65.8 to −36.8, *p* < 0.001). In Phe508del/Phe508del patients, LCI at baseline was 10 (IQR: 7.5–12.7) and after ELX/TEZ/IVA treatment it decreased to 8 (IQR: 6.3–10.1, *p* < 0.001), with a relative improvement of 15.3%. However, in those pretreated with TEZ/IVA or LUM/IVA LCI, the decrease was only 1.1 (IQR: −2.5 to −0.4, *p* < 0.001). Moreover, ppFEV_1_ improved by 12.5 points (IQR 4.4–21.3, *p* < 0.001) and SCC decreased by 42 mmol/lt (IQR −59 to −30.9, *p* < 0.001) [21].

Zemanick et al. conducted a survey in order to assess the safety and efficacy of ELX/TEZ/IVA in children aged 6–11 years. Sixty-six children with at least one Phe508del allele participated in this open-label phase 3 study, in which they received ELX/TEZ/IVA for 24 weeks. Overall, the triple combination treatment led to an increase in ppFEV_1_ by 10.2 percentage points (95% CI 7.9–12.6, *p* < 0.001), an amelioration in LCI_2.5_ of −1.71 units (95% CI −2.11 to −1.30, *p* < 0.001), in SCC of −60.9 mmol/lt (95% CI −63.7 to −58.2, *p* < 0.001) and in CFQ-R RD score of 7 points (95% CI 4.7–9.2, *p* < 0.001), through to week 24. The between sub-groups analysis (according to participants’ genotype) showed a similar increase in ppFEV_1_, LCI_2.5_ and CFQ-R RD score. In contrast, the SCC improvement was more important in the Phe508del homozygous participants (−70.4 mmol/lt, 95% CI −75.6 to −65.3, *p* < 0.001) than in the Phe508del/MF patients (−55.1 mmol/lt, 95% CI −59 to −51.2, *p* < 0.001) [22].

Petersen et al. designed a study cohort in which they found that adults with CF treated with ELX/TEZ/IVA presented an annual rate of increase in ppFEV_1_ of 7.81 points/year (95% CI 6.39–9.23, *p* < 0.0001) [23].

Korten et al., in their observational pilot study, observed that the 16 patients who started ELX/TEZ/IVA treatment had an improvement in their lung function, the LCI decreased to 6.84 (IQR 6.39–7.89, *p* < 0.003), FEV_1_ z-score increased to −0.39 (IQR −1.08 to 0.4, *p* < 0.007) and FEV_1_/FVC z-score to 0.31 (IQR −0.33 to 0.78, *p* < 0.0009), from baseline. SCC improved to 51 mmol/lt (IQR 32–59 mmol/lt, *p* < 0.002) compared to SCC before treatment initiation [24].

**Growth and nutrition**

CFTR modulators have a beneficial effect on growth and weight gain, as shown by different studies. DiMango et al. observed that BMI was increased from 21.8 kg/m^2^ (95% CI 21–22.6) to 22.7 kg/m^2^ (95% CI 21.8–23.6, *p* < 0.001) in CF patients with at least one Phe508del allele after 3 months of triple combination treatment [18]. Furthermore, as noticed by Nichols et al. after 6 months of ELX/TEZ/IVA therapy in patients with at least one Phe508del allele, there was a BMI increase by 1.2 kg/m^2^ from baseline for adults (95% CI 1.05–1.44) and 0.3 z-score in adolescents. Amelioration in BMI was similar in all subgroups, regardless of previous treatment [19].

After 8–16 weeks of the triple treatment, Graeber et al. observed an increase in BMI by 1.1 kg/m^2^ (IQR 0.4–1.9, *p* < 0.001) in Phe508del/MF, and by 1.2 kg/m^2^ (IQR 0.5–1.5, *p* < 0.001) in Phe508del/Phe508del patients pretreated with TEZ/IVA. Another study conducted by Graeber et al. showed that Phe508del/MF patients increased their BMI by 0.8 kg/m^2^ (IQR 0.3–1.8, *p* < 0.001) and Phe508del/Phe508del patients by 0.7 kg/m^2^ (IQR 0–1.3, *p* < 0.001) after ELX/TEZ/IVA treatment initiation [20].

Zemanick et al. noticed that BMI, BMI-for-age z-score, weight, weight-for-age z-score, and height, in children 6–11 years old with at least one Phe508del mutation, improved over the 24 weeks of ELX/TEZ/IVA treatment [22]. Petersen et al. conducted a single-center, retrospective, observational study with 134 adults with CF in order to assess the impact of ELX/TEZ/IVA treatment on body weight after 12.2 months of follow-up. The annual BMI change was 1.47 kg/m^2^/year (95% CI 1.08–1.87, *p* < 0.001) and the annual increase in body weight was 4.43 kg/year (95% CI 3.14–5.36, *p* < 0.001). It was noted that CF patients with pancreatic insufficiency had a more significant improvement in BMI after the triple therapy initiation compared to those with pancreatic sufficiency. Furthermore, the rate of BMI change of underweight patients and those with normal weight was decreased (from 7.5% to 2.2% and 65.7% to 56.7%, *p* < 0.001, respectively). On the contrary, the researchers observed an increase in the rate of BMI change of overweight patients from 19.4% to 31.3% (*p* < 0.001) and in obesity from 7.5% to 9.7%, *p* < 0.001) [23].

Scully et al., in a prospective observational study with 23 patients and at least one Phe508del allele, had a ppFEV_1_ 79 ± 5% at baseline, which increased to 91 ± 5% after ELX/TEZ/IVA treatment (*p* < 0.0001) and ppFVC 92 ± 4% at initiation, which ameliorated to 99 ± 5% (*p* < 0.0005) after treatment [25].

**Other parameters**

i. Cardiometabolic parameters

Petersen et al. studied the effect of ELX/TEZ/IVA treatment on different cardiometabolic parameters. They observed that adults with CF without CF-related diabetes (CFRD) presented an annual decrease in random blood glucose of 0.78 mM/year (95% CI −0.23 to −1.33, *p* < 0.01) and in HbA_1_c of 0.16%/year (95% CI −0.07 to −0.26, *p* < 0.005). Furthermore, in adults with CFRD, an annual improvement in HDL of 0.23 mM/year (95% CI 0.04–0.42, *p* < 0.05) and an annual increase in total cholesterol of 0.67 mM/year (95% CI 0.37–0.97, *p* < 0.0005) and in LDL of 0.47 mM/year (95% CI 0.25–0.69, *p* < 0.0005) was noticed. As for blood pressure, the annual increase in systolic blood pressure (SBP) was 4.94 mmHg (95% CI 0.31–9.57, *p* < 0.05), and in diastolic blood pressure (DBP) 3.49 mmHg (95% CI 0.65–6.34, *p* < 0.05). At baseline, 35% of participants met the criteria for stage 1 or stage 2 hypertension, and 65% at the end of the study (*p* < 0.0001) [23].

ii. Glycemic status

Researchers investigated the effect of the triple combination treatment on glucose tolerance and CFRD, given the fact that its impact on this domain is not well understood. Scully et al. performed a prospective, observational study with 34 adults with at least one Phe508del mutation and a history of exocrine pancreatic insufficiency, in which they used continuous glucose monitoring (CGM) sensors for 14 days before ELX/TEZ/IVA initiation and 3–12 months after therapy, with the purpose of assessing the effect of the triple CFTR modulator treatment on glycemic status (50% of the participants had already had CFRD). Overall, after the ELX/TEZ/IVA treatment, the CGM average glucose diminished to 124 ± 8 mg/dL from 136 ± 9 mg/dL (*p* < 0.018), percentage time >200 mg/dL decreased from 16.4 ± 4.1 to 9.7 ± 2.6 (*p* < 0.006) and the peak sensor value decreased to 280 ± 20 mg/dL from 306 ± 21 mg/dL (*p* < 0.045). No significant changes were noticed in measures of hypoglycemia, weight, or BMI. In subgroup measurements between candidates with and without CFRD, percentage time >200 mg/dL was significantly decreased in both groups. In patients with CFRD CGM, average glucose (AG) improved from 162 ± 10 to 144 ± 10 mg/dL (*p* < 0.033), % time 70–180 mg/dL from 63.6 ± 5.7 to 73.5 ± 5.3 mg/dL (*p* < 0.011) and weight increased from 67.7 ± 3.6 kg to 73.3 ± 3.6 kg (*p* < 0.045) after ELX/TEZ/IVA treatment [25].

Korten et al. carried out an observational pilot study with 16 CF patients ≥12 years with at least one Phe508del allele and without known CFRD, in whom they performed an oral glucose tolerance test (OGTT) before and 4 to 6 weeks after the start of ELX/TEZ/IVA treatment. Patients carried out a CGM system 3 days before till 7 days after initiation of the therapy. OGTT improved after the treatment (*p* < 0.02). Before treatment, five patients were categorized into normal glucose tolerance (NGT) group, two into intermediate glucose tolerance (INDET) group, six into impaired glucose tolerance (IGT) and two into CFRD group. After ELX/TEZ/IVA treatment, nine participants were classified in NGT category, four into INDET, two into IGT and there were no participants in the CFRD category. In addition, 60 min, 90 min, and 120 min plasma glucose significantly decreased after treatment (*p* < 0.03, *p* < 0.04, and *p* < 0.03, respectively); on the contrary, no changes were observed in fasting plasma glucose and in 180 min OGTT plasma glucose. Levels of 120 min insulin, 180 min insulin, and 180 min plasma C-peptide were improved under ELX/TEZ/IVA treatment (*p* < 0.01, *p* < 0.006, and *p* < 0.005, respectively). The area under the curve (AUC) was lower for blood glucose and blood insulin (*p* < 0.008, and *p* < 0.02) after ELX/TEZ/IVA initiation. No difference occurred in HbA_1_c with the triple combination therapy. As for the CGM results, the researchers noticed no difference in glucose levels before and after the triple treatment therapy [24].

Piona et al. performed a prospective, observational study with 21 CF patients aged ≥6 years, in order to assess the β-cell function (estimated by the derivative control (DC) = the response of β-cells to glucose increase, and by the proportional control (PC) = the response of β-cells to glucose concentration), insulin clearance and insulin sensitivity after CFTR modulator treatment. A total of five of the 21 patients, who had at least one Phe508del allele and severe lung disease (FEV_1_ < 40%), had a spirometry, a SC test, and an OGTT 1–12 weeks before and 12–18 months after ELX/TEZ/IVA treatment. The results showed no significant change in β-cell DC, PC, insulin clearance, and insulin sensitivity after the triple therapy. Only HbA_1_c was significantly reduced in these patients (*p* = 0.04) [26].

**Advanced Lung Disease (ALD)**

CF patients with advanced lung disease (ppFEV_1_ < 40%) are usually excluded from clinical trials. However, given the very promising results of ELX/TEZ/IVA, researchers conducted studies that included patients with ALD. Stylemans et al. conducted a real-life follow-up study, in which they included 14 adult patients, Phe508del homozygous with ppFEV_1_ < 30% or Phe508del/Minimal Function mutation genotype and with ppFEV_1_ < 40%. Participants had a median age of 36 years, the majority of whom had a Phe508del/Phe508del genotype, all were diagnosed with pancreatic insufficiency and 50% had CFRD. After 4 weeks of treatment ppFEV_1_ increased by 12 percentage points (*p* < 0.001) and remained stable thereafter. Median ppLCI decreased by 31% (*p* = 0.002) from baseline. Taking into consideration the significant amelioration of other markers, such as acinar ventilation heterogeneity, RV/TLC ratio, FVC, and V_A_, they concluded that there was a considerable ventilation distribution improvement. In addition, the exacerbation rate diminished from 0.33 to 0.07/month after 3 months of treatment [27].

Bermingham et al. performed a retrospective cohort study with 50 adult CF patients with at least one copy of the Phe508del mutation and ALD. The participants’ median age was 32 ± 8.2 years, with a ppFEV_1_ < 40%, all had pancreatic insufficiency, 54% had CFRD, 38% presented high risk characteristics (such as a history of pneumothorax, massive hemoptysis or colonization with nontuberculous mycobacteria or Burkholderia spp) and 40 participants had a referral for lung transplantation. The researchers noticed that, after 39.1 ± 24.8 days of ELX/TEZ/IVA treatment, the ppFEV_1_ increased from baseline by 7.9 percentage points (95% CI 5.85–10.2, *p* < 0.0001), and the ppFVC by 10.5% (95% CI 7.76–13.48, *p* < 0.0001), with no significant difference in ppFEV_1_ between Phe508del homozygous and Phe508del heterozygous patients (increase in ppFEV_1_ by 7.1 ± 6.0 and 9.5 ± 10.0, *p* = 0.29, respectively) [28].

Martin et al., using questionnaires, assessed the quality of life of patients ≥ 12 years old (median age: 35 years), having the following CFTR genotypes: Phe508del/Phe508del, Phe508del/other or unreported, and with ALD (ppFEV_1_ < 40% and/or an indication for lung transplantation), after initiation of ELX/TEZ/IVA treatment. In general, after 4.3 (3.0–5.6) months of the triple combination treatment, patients reported an amelioration of pulmonary symptoms, a decrease in cough, sputum production, pulmonary exacerbations, IV antibiotics use, time spent on chest physiotherapy and suspension of lung transplantation discussions. They also reported an improvement in extrapulmonary symptoms, such as sleep quality and appetite, an amelioration in glycemic control, or increase in self-esteem [29].

Burgel et al., in a prospective observational study, assessed the impact of ELX/TEZ/IVA treatment in 245 patients aged ≥ 12 years (median age = 31 years) with at least one copy of the Phe508del mutation and ppFEV_1_< 40%, and/or being on the lung transplant waiting list or under transplantation evaluation. At 73 (32–88) days of ELX/TEZ/IVA treatment, ppFEV_1_ increased by 15.1% (95% CI 13.8–16.4, *p* < 0.0001), with no significant difference in ppFEV_1_ increase between those previously treated with a CFTR modulator and those not. Furthermore, ppFEV_1_ had a greater increase in those without oxygen and/or non-invasive ventilation (NIV) at the beginning of the treatment. Specifically, ppFEV_1_ increased by 16.2 points (95% CI 14.5–17.9, *p* < 0.0001) compared with those with oxygen and/or NIV before treatment (amelioration by +13.6%, 95% CI 11.6–15.7, *p* < 0.0001). As far as weight is concerned, this increased by 4.2 kg (95% CI 3.9–4.6, *p* < 0.0001), with a more significant increase in those not treated previously with a CFTR modulator (+4.5 kg, 95% CI 4.1–4.9, *p* < 0.0001) than those treated with another CFTR modulator (+3.4 kg, 95% CI 2.7–4, *p* < 0.0001). The ELX/TEZ/IVA therapy led to a decrease in other treatments; more specifically the number of patients who needed oxygen or enteral tube feeding was diminished by 50% (*p* < 0.001) and those in need of NIV by 30% (*p* < 0.001) [30].

The retrospective cohort study conducted by Carnovale et al. included 47 patients. All were aged ≥12 years (median age = 32 years old), had Phe508del/Minimal Function genotype, their ppFEV_1_ was <40%, or they were on the waiting list for lung transplantation. Lung function was evaluated after 1 and 6 months of ELX/TEZ/IVA treatment. The ppFEV_1_ increased by 10.69 percentage points (95% CI 8.05–13.33, *p* < 0.0001) and by 14.16 points (95% CI 11.43–16.89, *p* < 0.0001), after 1 and 6 months, respectively, from the baseline. The SCC decreased from a baseline of 91.1 mmol/lt to 52 mmol/lt (*p* < 0.00001) and to 46.2 mmol/lt (*p* < 0.001), after 1 and 6 months of treatment, respectively. CFQ-R RD score was ameliorated from a baseline of 55.5 to 83.3 (95% CI 50–100, *p* < 0.00001) after 1 month and to 91.6 (95% CI 61.1–100, *p* < 0.00001) after 6 months of therapy. A decrease of 77% in the annual rate of pulmonary exacerbations was reported due to the ELX/TEZ/IVA treatment and only one patient needed a single cycle of IV antibiotics. As for growth, the mean BMI at initiation was 20.7 kg/m^2^, which improved significantly to 21.4 kg/m^2^ (*p* = 0.00005) and to 22.6 kg/m^2^ (*p* < 0.00001) after 1 and 6 months of treatment, respectively [31].

As already known, ELX/TEZ/IVA treatment is safe and efficacious for patients ≥12 years old with at least one Phe508del mutation. Unfortunately, sometimes advanced lung disease is developed in early childhood, so there are children aged 6–11 years with a ppFEV_1_ < 40%, requiring intense treatment or lung transplantation. Salvatore et al. evaluated the effectiveness and safety of the triple therapy after 24 weeks of treatment in this group of patients. In this retrospective, observational study, nine children aged 6–11 years, with a median age of 9.75 years, Phe508del homozygous or with a Phe508del/MF genotype, and with ppFEV_1_ < 40%, participated. All had pancreatic insufficiency. The results showed a mean increase in ppFEV_1_ of 22.4 points (95% CI 15.22–29.52, *p* < 0.001), an improvement in ppFVC of 19.27 points (95% CI 14.38–24.16, *p* < 0.001) and in ppFEF_25–75_ of 26.7 (95% CI 15.61–37.79, *p* < 0.001) points from the baseline, after 24 weeks of treatment. The mean absolute change of BMI for age z-score was 0.60 (95% CI 0.33–0.87, *p* < 0.001), of weight for age z-score 0.41 (95% CI 0.20–0.62, *p* < 0.001) and of height for age z-score 0.31 (95% CI 0.05–0.57, *p* < 0.02), from baseline at week 24. The SCC significantly decreased by 74.8 (95% CI −62.44 to −87.07, *p* < 0.001) and the CFQ-R RD score increased to 100 (95% CI 100–100, *p* < 0.001) from the baseline value, over the 24 weeks of the study. Finally, an 80% lower rate of antibiotic use and zero hospitalizations were observed after 24 weeks of ELX/TEZ/IVA treatment [32].

Carnovale et al. conducted a 48-week retrospective observational study with 26 adult patients (median age was 31.1 years), Phe508del homozygous, and with ALD (ppFEV_1_ < 30% or ppFEV_1_ < 40%, rapidly declining and/or ≥6 PEx in the last year). All of these patients had pancreatic insufficiency and 42.3% were diagnosed with CFRD. The mean absolute increase in ppFEV_1_ was 14.48 points (95% CI 10.64–18.32, *p* < 0.0001), and in ppFVC 18.50 (95% CI 13.64–23.35, *p* < 0.0001) points, over 48 weeks of treatment. Moreover, BMI was improved by 2.08 kg/m^2^ (95% CI 1.63–2.52, *p* < 0.0001), the CFQ-R RD score increased by 32.6 points (95% CI 24.6–40.1) compared to baseline, the SCC decreased to 29.2 mmol/lt (*p* < 0.0001), and the rate of pulmonary exacerbations diminished by 97%, through 48 weeks of triple therapy [33].

Finally, Piona et al., in their observational pilot study, noted that five of the 21 participants, aged 22 ± 7.4 years, with Phe508del/Other genotype, and ALD (ppFEV_1_ < 40%) had an increase in ppFEV_1_ to 49 ± 11.27 (*p* < 0.041), FVC to 4.17 ± 0.95 lt (*p* < 0.014) and a decrease in SCC to 30.33 ± 11.72 mmol/lt (*p* < 0.002) from baseline, after 12–18 months of ELX/TEZ/IVA treatment [26].

**Lung transplant perspectives**

The CF patients included in the Bermingham et al. study with ALD had a history of lung transplant evaluation, but after the triple combination treatment seven patients were recategorized because they no longer met the criteria for lung transplantation due to their lung function improvement [28]. Similar results were found by Martin et al., as CF patients with ALD were taken off the lung transplantation candidate list after ELX/TEZ/IVA treatment [29]. Furthermore, 11 of 15 patients with severe lung disease who were on the transplantation list, after ELX/TEZ/IVA treatment were suspended, and 36 of 37, who were under evaluation for joining the list, no longer met the criteria for lung transplantation, according to the study conducted by Burgel et al. [30]. Another three patients in the study organized by Carnovale et al. decided to be removed from the lung transplantation waiting list after 6 months of ELX/TEZ/IVA treatment [31].

#### 3.2.2. Safety of Elexacaftor-Tezacaftor-Ivacaftor

Zemanick et al. assessed the safety of ELX/TEZ/IVA treatment in children 6–11 years old, in whom 98.5% of the participants had at least one AE, 54.5% of which were mild and 42.4% moderate in severity. The most common AEs experienced by the candidates were cough (42.4%), headache (24.2%), and pyrexia (21.2%), 10.6% of the children presented elevated levels of aminotransferases greater than three times the upper normal range, and in 1.5% the levels were greater than five times the upper normal limit. As far as rashes are concerned, 24.2% of the patients had mild or moderate rash events (e.g., rash erythematous, rash maculopapular, rash papular, skin exfoliation, or urticaria). Due to the development of a rash after the first dose of the triple treatment, the drug was discontinued in one child. CPK levels were not greater than five times the upper normal limit. No AE of hypertension were noticed, the systolic blood pressure changed from −1.4 mmHg to 0.4 mmHg, and diastolic blood pressure from −0.3 mmHg to 1 mmHg, through the 24 weeks of treatment [22].

Researchers were concerned with the safety of triple combination therapy in CF patients with ALD. Stylemans et al., during the 3-month therapy, showed that it was well-tolerated and only one patient had a drug-induced liver injury leading to treatment discontinuation. No rash, dyspnea, or cough was observed [27]. A serious adverse event of pancreatitis and distal intestinal obstructive syndrome occurred in one patient who participated in the Bermingham et al. study, without interruption of the treatment; mild side effects, such as rashes, constipation, and hypoglycemia were seen in 10 participants [28]. Burgel et al. reported some side effects, generally mild, such as localized (7.2%) or generalized rashes (3.8%), headache (4.2%), gastrointestinal symptoms (10.2%), and myalgia (4.7%). Elevated levels of SGPT ≥ 3 times the upper limit of normal were noticed in 2.5% of patients and elevated levels of SGOT ≥ 3 times the upper limit of normal in 0.8%. Increased bilirubin (≥3 times the upper normal) was seen in 4.7% of participants (three of whom already had cirrhosis) and elevated CPK levels greater than three times the upper normal limit occurred in 3.4% of the patients. None needed to discontinue the triple combination therapy [30].

The above mentioned results are summarized in Table 2.

## 4. Discussion

The CFTR modulator ELX/TEZ/IVA has been shown to be effective in Phe508del homozygous or Phe508del heterozygous with a minimal function mutation. The total number of included studies in this systematic review sums up the beneficial effects of this new medication.

Impaired lung function with a gradual deterioration, starting early in life, is a key feature of CF and ELX/TEZ/IVA improves pulmonary function significantly. Both clinical trials and observational studies highlight a substantial increase in FEV_1_, both in adults and children, after the initiation of treatment. LCI_2.5_, which is used to determine the ventilation inhomogeneity, also showed noteworthy improvements, when it was used as a surrogate of pulmonary function in studies. As for SCC, which is a clinical marker of CFTR function and is related to disease severity, the triple combination therapy showed that it can reduce its values even <30 mmol/lt.

The CFQ-R is a disease-specific health-related quality of life measure for children, adolescents, and adults with CF. It consists of 12 different domains, one of which concerns respiratory symptoms, which can be used in studies in order to assess the effect of the new therapies on the quality of life of CF patients. An increase in the CFQ-R RD score was observed with the new treatment. The use of ELX/TEZ/IVA also decreased the number of pulmonary exacerbations [34,35].

As far as metabolic parameters are concerned, ELX/TEZ/IVA promotes weight gain with an uncertain, but probably multi-factorial, mechanism. The new CFTR modulator ameliorates the appetite and augments food intake and weight gain. ELX/TEZ/IVA may rehabilitate part of the exocrine pancreatic function, leading to better intestinal absorption and weight gain. The increase in life expectancy in CF patients under the triple treatment results in a rising risk of hyperlipidemia and hypertension and is likely to increase the incidence of cardiovascular and cerebrovascular events [23].

The exact mechanism of CFRD and the role of CFTR modulators therapy in the pathophysiology of CFRD are not yet well defined. It is known that the dysfunction of insulin secretion causes CFRD. Even though CFTR protein is detected in pancreatic α and β-cells, implying a role in insulin secretion, this finding has not been confirmed by all studies. Indeed, minimal CFTR mRNA expression and CFTR protein were found in human islet cells. The impact of CFTR modulators on CFRD seems to be indirect; CFTR restoration could reduce the systemic and localized islet inflammation and, as a consequence, improve islet function and insulin sensitivity. In addition, a second possibility is the increased excretion of incretins from the gastrointestinal neuroendocrine cells, which enhance insulin secretion. On the other hand, improved calorie intake and intestinal absorption, due to CFTR modulator therapy, leads to weight gain and, as a result, increased insulin resistance. No clinical trials have evaluated the effect of ELX/TEZ/IVA on CFRD and glucose metabolism [36]. An observational study conducted by Scully et al. noticed an improvement in measures of hyperglycemia and glycemic variability with CGM after ELX/TEZ/IVA initiation [25]. Moreover, Korten et al. observed an improvement in endocrine pancreatic function with the triple modulator treatment as glucose tolerance for OGTT was improved [24].

Recent data on ADL in CF patients treated with ELX/TEZ/IVA have been very limited, because these patients are excluded from many studies, due to the adverse events and the unimportant lung function improvements noticed with previous CFTR modulators. However, positive events have been observed in small studies conducted in patients with ppFEV_1_ < 40%. The results were encouraging, as ELX/TEZ/IVA treatment ameliorated the ppFEV_1_, the CFQ-R RD, and the BMI and diminished the SCC in adults and children. It is very important that, after initiation of the treatment, many patients with ALD no longer met the criteria for lung transplantation.

In general, as has been demonstrated by clinical trials and observational studies, ELX/TEZ/IVA is a well-tolerated medication in all the CF subgroups. Patients included in the aforementioned studies mostly suffered from mild to moderate adverse events. The most frequent were cough, increased sputum production, pulmonary exacerbations, hemoptysis, and pyrexia. It was found that the increased fluidity of respiratory mucus after the ELX/TEZ/IVA initiation is responsible for cough and sputum production aggravation. Rashes (erythematous, maculopapular, papular, skin exfoliation, or urticaria) were a common side effect that occurred approximately in 4–10% of patients treated with ELX/TEZ/IVA. The exact mechanism of this event is unclear. Most likely, skin eruptions are mild to moderate in severity, but some important rashes may demand discontinuation of the triple CFTR modulator treatment. Elevations in blood pressure were noticed [10,23]. The role of CFTR modulator in vascular homeostasis is not known, as the direct effect of ELX/TEZ/IVA on non-epithelial cells is not yet understood. It has been hypothesized that CF patients have lower blood pressure because of the salt losses in their sweat, and consequently the improvement of CFTR function with the treatment leads to less salt wasting and higher levels of blood pressure [37,38,39].

Elevated levels of aminotransferases were noted in adults and children with CF and this was a common AE, which did not lead to treatment discontinuation. CPK levels were also found to increase, mostly in association with exercise. As far as gastrointestinal side effects of ELX/TEZ/IVA are concerned, one serious adverse event is distal intestinal obstruction syndrome (DIOS), which is mentioned in the clinical trial conducted by Middleton et al. [10]. It is already known that CF patients suffer from constipation, due to the thick intestinal mucus, intestinal mobility disorders, and indigested food being attached to the intestinal walls. After treatment initiation, the amelioration of the mucus hydration leads to fecal detachment of the bowel wall and its movement through the intestine, increasing the possibility of DIOS. In addition, the viscous bile with abnormal pH causes bile salts accumulation and bile gallstones formation, though the CFTR function restoration in the biliary epithelium, with the new treatment, leads to increased bile fluidity that could trigger the movement of pre-existing gallstones and subsequently the appearance of biliary colic and/or acute or chronic cholecystitis [37,40,41,42,43]. Testicular pain is another side effect described by Rotolo et al., as the thinner mucus blockage moves from either testes, vas deferens, or both when electrolytes balance is achieved with CFTR restoration [44]. In addition, Miller et al. described two cases with both papilledema and hypervitaminosis A after ELX/TEZ/IVA treatment, which was related to the elevated absorption of vitamin A supplements; given the known correlation of vitamin A and increased intracranial pressure (ICP), they supposed that hypervitaminosis A led to increased ICP [45]. Moreover, mental health issues, such as anxiety, depression, psychotic disorder, or obsessive-compulsive disorder, were reported by Barry et al. and Sutharsan et al. [12,14]. The exact mechanism of action remains unclear; it is probably related to the fact that this medication has a lipophilic nature; it passes through the blood–brain barrier and interacts with the CFTR or the serotonin receptor 5-hydroxytryptamine_2c_ (5-HT_2c_), which has been associated with anxiety, depression, and suicidality. Even though IVA has a high to moderate affinity for the 5-HT_2c_ receptor, suggesting that this CFTR modulator has a positive action in the behavior, the adverse events observed pose the question as to whether ELX and TEZ have a serotonergic effect, which contradicts the positive effect of IVA [46,47].

Drug-induced liver injury (DILI) led to discontinuation of the ELX/TEZ/IVA treatment in one patient with CF and ALD, according to Stylemans et al. DILI is defined by one of the following thresholds: (i) ALT ≥ 5 times the upper normal limit, (ii) ALP ≥2 times the upper normal limit with an elevation of γ-GT or (iii) ALT ≥ 3 times the upper normal limit and elevation of TBIL ≥ 2 times the upper normal limit. DILI can be intrinsic (dose-dependent, with an immediate effect, hours to days) or idiosyncratic (not dose-related and with a variable latency of onset of days to weeks). Interruption of the treatment is the intervention of choice in this case, but in critical patients with the need for non-replaceable drugs, favorable results have been noticed [48].

The development of the new CFTR modulators changed the natural course of the disease. Patients carrying at least one allele of the Phe508del mutation are eligible for the new therapies. Even though it is the most common CFTR mutation, with 85.5% of patients worldwide having at least one copy (44.1% are Phe508del homozygous and 41.4% are Phe508del heterozygous), there is a 14.5% of patients without any Phe508del mutation or with an unknown mutation [24]. This is the reason that individualized treatment is necessary. In this direction, international studies using intestinal organoids or nasal cells of CF patients and rare mutations, in order to assess their response to the novel molecules, are in progress. Research has demonstrated that results derived from ex vivo testing correlate fairly well with in vivo therapeutic changes and can be used as predictors of the clinical effectiveness of new drugs [49,50,51,52,53].

## 5. Conclusions

The new CFTR modulator has many positive effects on CF patients with at least one Phe508del allele, on their lung function, nutrition, quality of life, glycemic status, and other parameters. Even patients with ALD can benefit from the ELX/TEZ/IVA treatment and many have been suspended from the waiting list for lung transplantation. It is also proven that this new triple CFTR treatment is well tolerated with a favorable safety profile in all the subgroups of patients. Further studies are needed to establish the effect of this treatment on the rest of the CF manifestations (for example glycemic status, pancreatic insufficiency, fertility). As this new CFTR modulator is highly effective for patients with Phe508del mutations, a question has been raised as to whether it could be used in patients with rare CFTR mutations who have no indication for CFTR modulator treatment [54]. In this direction, studies with nasal cells and intestinal organoids from patients with rare mutations are in progress.

## Figures and Tables

**Table 1:** Summary of characteristics and results of case-control studies examing the effect of Elexacaftor/Tezacafor/Ivacaftor on different parameters in individuals with Cystic Fibrosis.

Trial	Study Population	Intervention Analyzed	Primary Outcome
Keating et al. (2018) Randomized, placebo–controlled, double-blind, phase 2 trial [9]	Phe508del heterozygous with a MF (n = 95) and Ph508del homozygous (n = 28), after a 4 week TEZ/IVA run in, aged ≥18 years	- Phe508del/MF (n = 21): VX-445 200 mg × 1 + TEZ 100 mg × 1 + IVA 150 mg × 2 daily - Phe508del/Phe508del (n = 21): VX-445 200 mg × 1 + TEZ 100 mg × 1 + IVA 150 mg × 2 daily	- Safety at 4 weeks: 92% of patients received VX-445 + TEZ + IVA had AE: 53% mild events, 43% moderate and 4% serious, 100% of patients received placebo had AE - FEV1 % (at 4 weeks): Phe508del/MF Increase in FEV_1_%: 13.8 points (95% CI 10.9–16.6) Phe508del/Phe508del Increase in FEV_1_: 11.0 points (95% CI 7.9–14.0)
Middleton et al. (2019) Randomized, placebo–controlled, double-blind, phase 3 trial [10]	Phe508del heterozygous with a MF, aged ≥12 years, FEV_1_ 40–90%, stable disease during the 4 week screening period before the beginning of the triple combination or the placebo (n = 403)	ELX 200 mg × 1 + TEZ 100 mg × 1 + IVA 150 mg × 2 daily (n = 200)	Increase in FEV_1_%: 13.8 points at 4 weeks (95% CI 12.4–15.4)
Heijerman et al. (2019) Multi-centre, randomized, active-controlled, double-blind, phase 3 trial [11]	Phe508del homozygous, aged ≥12 years, FEV_1_ 40–90% with stable disease, as judged by the investigators (n = 113)	ELX 200 mg × 1 + TEZ 100 mg × 1 + IVA 150 mg × 2 daily (n = 55)	Increase in FEV_1_% by 10 points (95% CI 7.4–12.6) at week 4
Barry et al. (2021) Phase 3, double-blind, randomized, active-controlled trial [14]	Phe508del/Gating mutation or Phe508del/ Residual mutation, aged ≥12 years (n = 258)	ELX 200 mg × 1 + TEZ 150 mg × 1 + IVA 150 mg × 2 daily (n = 132)	Change from baseline in FEV_1_% at week 8: 3.7 points (95% CI 2.8–4.6), higher by 3.5 points (95% CI 2.2–4.7) relative to active control
Sutharsan et al. (2022) Multicentre, double-blind, active-controlled, phase 3b trial [12]	Phe508del homozygous, aged ≥12 years, FEV_1_% 40–90, with stable disease (n = 175)	ELX 200 mg × 1 + TEZ 100 mg × 1 + IVA 150 mg × 2 daily (n = 87)	Absolute change in CFQ-R RD from baseline at week 24: 17.1 (95% CI 14.1–20.1)
Mainz et al. (2022) Prospective study [16]	Phe508del homozygous or heterozygous, aged ≥18 years (UK cohort) or ≥12 years (German cohort) (n = 152)	ELX/TEZ/IVA combination (n = 107): 60 PwCF under a different CFTR modulator before 47 PwCF without previous treatment	Decrease in total CFAbd-Score at week 24 by 29% during treatment (*p* < 0.01)
Miller et al. (2022) Case-crossover analysis [17]	PwCF who were treated by ELX/TEZ/IVA before 1/12/2019 (n = 778)	ELX/TEZ/IVA (n = 389)	- Change in days with a health care visit: −2.5 (95% CI −3.31, −1.7) - Change in inpatients visits: −0.16 (95% CI −2.2, −1) - Decrease in days with an infection-related visit: −0.62 (−0.93, −0.31) - Decrease in distinct antibiotics prescribe: −0.78 (−1.03, −0.54)
Mall et al. (2022) Phase 3b, randomized, double blind, placebo-controlled study [15]	Phe508del heterozygous with a MF, aged 6–11 years (n = 121)	- Children < 30 kg: ELX 100 mg × 1 + TEZ 50 mg × 1 + IVA 75 mg × 2 daily - Children ≥ 30 kg: ELX 200 mg × 1 + TEZ 100 mg × 1 + IVA 150 mg × 2 daily (n = 60)	Decrease in LCI_2.5_ from baseline at week 24: 2.29 units (95% CI 1.97–2.6) and between groups difference −2.26 (95% CI −2.71 to −1.81)
Migliorisi et al. (2022) Case-control study [13]	PwCF with at least one Phe508del mutation and severe pulmonary disease (ppFEV_1_ < 40%) (n = 26)	ELX/TEZ/IVA	- Increase 10–15 points in ppFEV_1_ in treated patients - Amelioration in radiological findings - Increase in BMI of treated patients - 77% of case group patients presented a decrease in sweat chloride concentration - Increase in CFQ-R score in 100% of in case group patients - *P. aerigunosa* was detected in sputum of treated patients vs *S. aureus* in samples of the control group - Reduction in pulmonary exacerbations (*p* < 0.05) after 1 year of ETI treatment in case group patients

MF = minimal function mutation, TEZ = Tezacaftor, IVA = Ivacaftor, AE = adverse events, FEV_1_ = Forced expiratory volume in 1 s, ELX = Elexacaftor, RR = Rate ratio, BMI = Body Mass Index, ΕΤΙ = Elexacaftor/Tezacaftor/Ivacaftor, CFAbd-Score = Cystic-Fibrosis Abdominal Score, PwCF= People with Cystic Fibrosis, HC = Healthy Controls, LCI_2.5_ = Lung Clearance Index 2.5.

**Table 2:** Summary of characteristics and results of observational studies examing the effect of Elexacaftor/Tezacafor/Ivacaftor on different parameters in individuals with Cystic Fibrosis.

Study	Study Population	Primary Outcomes
DiMango et al. (2020) Prospective Cohort Study [18]	-Adults with CF with at least one copy of Phe508del mutation (n = 43)	- BMI improvement from 21.8 to 22.7 (*p* = 0.000002) - Increase in ppFEV_1_ from 65% to 76% (*p* = 0.0000005) - Improvement of all domains of CFQ-R
Stylemans et al. (2021) Real-life follow-up study [27]	-PwCF aged ≥18 years -Genotype Ph508del/Phe508del or Phe508del/MF -Severe lung disease and ppFEV_1_ < 30% for Phe508del/Phe508del genotype and <40% for Phe508del/MF genotype (n = 14)	- Increase in ppFEV_1_ by 12% at week 4 (*p* < 0.001) - Decrease in ppLCI by 31% from baseline at week 4 (*p* < 0.002) - Decrease in acinar Ventilation heterogeneity by 411% predicted at week 4 (*p* < 0.001) - Improvements in RV/TLC ratio to 0.49 % from 0.58% at week 4 (*p* < 0.001) - Increase in ppFVC by 13 points at 1 month (*p* < 0.001) - Increase in V_A_ to 4.14 lt from 3.64 lt at week 4 (*p* < 0.001) - Diminished exacerbation rate to 0.07/month from baseline at 3 months
Bermingham et al. (2021) Retrospective cohort study [28]	PwCF aged >18 years with advanced lung disease (n = 50)	- Increase in ppFEV_1_ of 7.9 points (95% CI 5.85–10.2) - Increase in ppFVC of 10.5 points (95% CI 7.76–13.48) - Fewer patients needed lung transplant planning
Martin et al. (2021) [29]	PwCF aged ≥ 12 years with advanced lung disease (ppFEV_1_ < 40% and/or indication for lung transplantation) (n = 110)	- Reduction of respiratory symptoms (cough, pulmonary exacerbations) - Improvement of appetite and sleep - Increase in gastrointestinal symptoms - Less time needed for other kinds of treatments (ex. chest physiotherapy) - Diminished use of antibiotics and less admissions in hospitals - Suspension from lung transplantation list - Amelioration of body self-esteem, self-confidence and autonomy
Burgel et al. (2021) Prospective Observational cohort study [30]	PwCF ≥12 years old, with at least one Phe508del mutation and advanced lung disease (ppFEV_1_ < 40 and/or under evaluation for transplantation (n = 245)	At 3 months: - Increase in ppFEV_1_ by 15.1 points (95% CI 13.8–16.4) - Increase in ppFEV_1_ by 16.2 (95% CI 14.5–17.9) for PwCF not treated with O_2_ or NIV - Increase in ppFEV_1_ by 13.6 points (95% CI 11.6–15.7) for those without O_2_ or NIV - Mean increase in BMI 4.2 kg (95% CI 4.1–4.9) - 50% reduction in O_2_ requirements (*p* < 0.001) - 30% decrease in NIV use (*p* < 0.001) - 50% decrease in enteral tube feeding (*p* < 0.001) - 2 out of 16 received a transplant • 56.4% decrease in lung transplantations (*p* = 0.002) • No change in number of deaths without transplantation
Zemanick et al. (2021) Phase 3, two-part, open-label, multicenter trial [22]	Children 6–11 years old with Phe508del/MF genotype or Phe508del homozygous (n = 66)	- 98.5% of children presented AE, 54.5% of which were mild and 42.4% moderate - PK: 30 kg is the weight limit for the administration of the full adult daily dose of ETI instead of the 50% of it - Through week 24: • Increase 10.2 points in ppFEV_1_ from baseline (95% CI 7.9–12.6) Change in CFQ-R RD score of 7 points (95% CI 4.7–9.2) Decrease in LCI_2.5_ by 1.71 units (95% CI −2.11 to −1.30, *p* < 0.001) Decrease of sweat chloride concentration by 60.9 mmol/lt (95% CI −63.7 to −58.2) BMI, BMI for age z-score, weight, weight for age z-score, height increased
Scully et al. (2021) Prospective single-center Observational study [25]	PwCF aged >18 years Phe508del heterozygous and with or without CFRD (n = 23)	- Decrease in AG (*p* < 0.018), SD (*p* < 0.001), % time Gly > 200 mg/dL (*p* < 0.006), peak sensor value (*p* < 0.45) - Increase in %time Gly 70–180 mg/dl (*p* < 0.04)
Carnovale et al. (2021) Retrospective Cohort Study [31]	PwCF aged >12 years with Phe508del/MF genotype and advanced lung disease (n = 47)	Increase 10.69% (95% CI 8.05–13.33) in ppFEV_1_ at week 4 and 14.16 points (95% CI 11.43–16.89) at week 24
Nichols et al. (2021) PROMISE STUDY Prospective Observational Study [19]	PwCF ≥ 12 years old with at least one copy of Phe508del At baseline 238 were on no CFTR treatment, 34 were on IVA and 215 on a two-drug modulator use (LUM/IVA or TEZ/IVA) (n = 487)	At 6 months: - Average increase in ppFEV_1_ by 9.8 points (95% CI 8.8–10.8) - Average decrease in SCC by 41.7 mmol/lt (95% CI −43.8, −39.6) - Increase in CFQ-R RD score by a mean of 20.4 points - Mean increase in BMI of 1.2 kg/m^2^ in adults and 0.3 z-score in children
Petersen et al. (2021) Single-center, retrospective, observation study [23]	Adults with CF, Phe508del heterozygous (pregnant and lung transplant patients the previous year or since starting the treatment were excluded) (n = 134)	- Increase in annualized difference in BMI by 1.47 kg/m^2^/year (95% CI 1.08–1.87) - Increase in annualized difference in weight by 4.55 kg/year (95% CI 3.14–5.36) - Increase in SBP by 4.94 mmHg/year (95% CI 0.31–9.57) - Increase in DBP by 3.49 mmHg per year (95% CI 0.31–9.57) - Decrease in protein gap by 5.84 g/L/year (95% CI −4.67 to −7.04) - In PWCF without CFRD: Decrease in annualized rate of random blood Gly by 0.78 mM/year (95%CI −0.23 to −1.33) Decrease in annualized rate of HbA_1_c by 0.16%/year (95% CI −0.07 to −0.26) - In PwCF with CFRD: Increase in total cholesterol by 0.67 mM/year (95% CI 0.37–0.97) Increase in LDL by 0.47 mM/year (95% CI 0.25–0.69) Increase in HDL by 0.23 mM/year (95%CI 0.04–0.42)
Salvatore et al. (2022) A 24-week Retrospective Observational Study [32]	Children aged 6–11 years with Phe508del/Phe508del or Phe508del/MF genotypes, and ppFEV_1_ < 40% (n = 9)	22.4 points increase in ppFEV_1_ at week 24 (95% CI 15.22–29.52)
Carnovale et al. (2022) A 48-week Retrospective Observational Study [33]	PwCF aged ≥ 6 years, Phe508del homozygous with LUM/IVA or TEZ/IVA treatment for at least 6 months and severe lung disease (n = 26)	- Increase in ppFEV_1_% from baseline by 12.06 points (95% CI 9.47–16.98) at week 4, by 13.22 points (95% CI 9.47–16.98) at week 12, by 15.32 points (95% CI 11.3–19.34) at week 24 and by 14.48 points (95% CI 10.64–18.32) at week 48 - Increase in ppFVC% from baseline by 13.08 points (95%CI 8.54–17.62) at week 4, by 14.59 points (95%CI 9.69–19.49) at week 8, by 18.89 points (95% CI 14.20–23.59) at week 24 and by 18.50 points (95% CI 13.64–23.35) at week 48
Graeber et al. (2022) Prospective Multicenter Observational Study [20]	PwCF ≥ 12 years old Phe508del homozygous or Phe508del/MF with no previous exposure to ELX/TEZ/IVA (n = 107)	Phe508del/MF: Decrease in SCC by 48.5 mmol/L (IQR −65.3 to −34.1) Phe508del/Phe508del: Reduction in SCC by 13 mmol/L (IQR −20.3 to −5) using TEZ/IVA and further decrease by 50.5 mmol/L (IQR −60.3 to −36.3) using ELX/TEZ/IVA treatment Reduction in SCC by 61 mmol/L (IQR −74 to −41) in those without previous therapy
Korten et al. (2022) Observation Pilot Study [24]	PwCF ≥ 12 years old with at least one copy of Phe508del mutation and with no CFRD (n = 16)	- OGTT improved after treatment (*p* < 0.02): 7 patients improved in OGTT 5 had a normal result 2 remained stable 1 changed from indeterminate glucose tolerance to impaired glucose tolerance - Improvement in plasma Gly levels at 60, 90 and 120 min (*p* < 0.03, *p* < 0.04, *p* < 0.03 respectively) - Lower insulin levels at 120 and 180 min (*p* < 0.01, *p*< 0.006 respectively) - Lower C-peptide levels at 120 and 180 min (*p* < 0.08 and *p* < 0.005 respectively) - HbA_1_c values were stable - Mean, minimum, maximum Gly levels and percentage of Gly level time appeared no difference after treatment
Piona et al. (2022) Prospective Observational Study [26]	PwCF ≥ 6 years old with at least one Phe508del mutation (n = 21)	12–18 months after treatment with ELX/TEZ/IVA: - No difference in glucose tolerance, beta-cell function, insulin clearance and insulin sensitivity

ELX/TEZ/IVA = Elexacaftor/Ivacaftor/Tezacaftor, BMI = Body Mass Index, PwCF = people with cystic fibrosis, ppFEV1 = percent predicted forced expiratory volume in 1 sec, ppLCI = percent predicted lung clearance index, FVC = forced vital capacity, NIV = non invasive ventilation, V_A_ = alveolar volume, RV = residual volume, TLC = total lung capacity, AG = average glucose, SD = standard deviation, Gly = Glucose, LUM = Lumacaftor, SCC = Sweat Chloride Concentration, SBP = Systolic Blood Pressure, DBP = Diastolic Blood Pressure, mM = Millimolar, CFRD = Cystic fibrosis related diabetes, CGM = Continuous Glucose Monitoring, PK = pharmacokinetics, PEx = pulmonary exacerbations, SCC = Sweat Chloride Concentration, OGTT = Oral Glucose Tolerance Test, RV/TLC = ratio of residual volume over total lung capacity, IQR = interquartile range.

## Abbreviations

5-HT_2c_	5-hydroxytryptamine_2c_
AE	Adverse Event
AG	Average Glucose
ALD	Advanced Lung Disease
ALP	Alkaline Phosphatase
ALT	Alanine Transaminase
AUC	Area Under the Curve
BMI	Body Mass Index
CF	Cystic Fibrosis
CFAbd-Score	Cystic Fibrosis Abdomen Score
CFQ-R RD	Cystic Fibrosis Questionnaire-Revised respiratory domain
CFRD	Cystic Fibrosis-related diabetes
CFTR	Cystic Fibrosis Transmembrane Conductance Regulator
CGM	Continuous Glucose Monitoring
CI	Confidence Interval
DA	Disorders of Appetite
DBM	Disorders of Bowel Movement
DBP	Diastolic Blood Pressure
DC	Derivative Control
DILI	Drug-induced liver injury
DIOS	Distal Intestinal Obstruction Syndrome
ELX/TEZ/IVA	Elexacaftor/Tezacaftor/Ivacaftor
ETI	Elexacaftor/Tezacaftor/Ivacaftor
FDA	Food and Drug Administration
FEV_1_	Forced Expiratory Volume in the 1st second
FVC	Forced Vital Capacity
GERD	Gastro-esophageal Reflux Disease
HbA_1_c	Hemoglobin A_1_c
HC	Healthy Controls
HDL	High-Density Lipoprotein
ICP	Intracranial Pressure
IGT	Impaired Glucose Tolerance
INDET	Intermediate Glucose Tolerance
IQR	Interquartile range
IVA	Ivacaftor
LCI	Lung Clearance Index
LCI_2.5_	Lung Clearance Index (lung volume turnover required to reach 2.5% of starting N_2_ concentration)
LDL	Low-Density Lipoprotein
LUM	Lumacaftor
MF	Minimal Function
mM	Millimolar (—mmol/lt)
NGT	Normal Glucose Tolerance
NIV	non-invasive Ventilation
OGTT	Oral Glucose Tolerance Test
PC	Proportional Control
PEx	pulmonary exacerbations
ppFEF_25–75_	percent predicted forced mid-expiratory flow rate
ppFEV_1_	percent predicted Forced Expiratory Volume in the first second
ppFVC	percent predicted Forced Vital Capacity
ppLCI	percent predicted Lung Clearance Index
PRISMA	Preferred Reporting Items for Systematic reviews and Meta-Analyses
PwCF	People with Cystic Fibrosis
QoL	Quality of Life
RR	Rate Ratio
RV/TLC	Residual Volume/Total Lung Capacity
SBP	Systolic Blood Pressure
SC test	Sweat Chloride test
SCC	Sweat Chloride Concentration
TBIL	Total Bilirubin
V_A_	Alveolar Volume
